# Electron Tomography Analysis of Thylakoid Assembly and Fission in Chloroplasts of a Single-Cell C4 plant, *Bienertia sinuspersici*

**DOI:** 10.1038/s41598-019-56083-w

**Published:** 2019-12-23

**Authors:** Keith Ka Ki Mai, Wai-Tsun Yeung, Sang-Yun Han, Xiaohao Cai, Inhwan Hwang, Byung-Ho Kang

**Affiliations:** 1 Centre for Cell and Developmental Biology, State Key Laboratory for Agrobiotechnology, School of Life Sciences, The Chinese University of Hong Kong, Shatin, New Territories, Hong Kong China; 2 Division of Integrative Biosciences and Biotechnology, Pohang University of Science and Technology, Pohang, 37673 Korea; 3 Mullard Space Science laboratory, Holmbury St. Mary Dorking, Surrey, RH5 6NT UK

**Keywords:** Cell biology, Plant sciences

## Abstract

*Bienertia sinuspersici* is a single-cell C4 plant species of which chlorenchyma cells have two distinct groups of chloroplasts spatially segregated in the cytoplasm. The central vacuole encloses most chloroplasts at the cell center and confines the rest of the chloroplasts near the plasma membrane. Young chlorenchyma cells, however, do not have large vacuoles and their chloroplasts are homogenous. Therefore, maturing *Bienertia* chlorenchyma cells provide a unique opportunity to investigate chloroplast proliferation in the central cluster and the remodeling of chloroplasts that have been displaced by the vacuole to the cell periphery. Chloroplast numbers and sizes increased, more notably, during later stages of maturation than the early stages. Electron tomography analyses indicated that chloroplast enlargement is sustained by thylakoid growth and that invaginations from the inner envelope membrane contributed to thylakoid assembly. Grana stacks acquired more layers, differentiating them from stroma thylakoids as central chloroplasts matured. In peripheral chloroplasts, however, grana stacks stretched out to a degree that the distinction between grana stacks and stroma thylakoids was obscured. In central chloroplasts undergoing division, thylakoids inside the cleavage furrow were kinked and severed. Grana stacks in the division zone were disrupted, and large complexes in their membranes were dislocated, suggesting the existence of a thylakoid fission machinery.

## Introduction

C4 photosynthesis is an adaptation in plants that suppresses photorespiration and increases efficiency of water and nitrogen use^1^. A key feature of C4 photosynthesis is the dimorphism of chloroplasts in photosynthetic tissues. One type of chloroplast concentrates atmospheric CO_2_ into a C4 acid intermediate. CO_2_ is regenerated in or near the other type of chloroplast where Rubisco brings the CO_2_ into the Calvin cycle. These differential functions lead to dissimilar energy requirements and ultrastructural features between the two classes of chloroplasts^2^.

In Kranz anatomy C4 plants, such as maize, the two classes of chloroplasts are segregated in mesophyll cells and bundle sheath cells^3^. In maize, an NADP-ME type C4 species, chloroplasts in mesophyll cells have higher concentrations of grana stacks because they contain photosystem II (PSII) associated with thylakoid stacking. Linear electron flow operates in the chloroplasts in mesophyll cells to produce both NADPH and ATP. Chloroplasts in bundle sheath cells have fewer stacked thylakoids because they contain less PSII and produce ATP through cyclic electron flow^4,5^.

It was assumed that C4 photosynthesis was synonymous with Kranz anatomy, but in the early 2000s, several species of single-cell C4 (SCC4) plants were discovered^3,4^. Unlike plants with Kranz anatomy, the two classes of chloroplasts in SCC4 plants are spatially segregated within a single cell and exhibit dimorphism related to varying abundances of PSII and photosystem I (PSI) in their thylakoids. At least four species, *Bienertia cycloptera*, *Bienertia sinuspersici*, *Bienertia kavirense*, and *Suaeda aralocaspica*, have been found to exhibit single-cell C4 photosynthesis^5^. *Bienertia sinuspersici* (*Bienertia*), a member of the family *Chenopodiaceae*, has become a model species in the characterization of SCC4 photosynthesis.

In *Bienertia*-type SCC4 plants, chlorenchyma cells have central chloroplasts (CCs) and peripheral chloroplasts (PCs). *Bienertia* CCs are located in a tightly packed central cytosolic compartment (CCC). PCs are dispersed around the outer edges of the cell near the plasma membrane. PCs generate a C4 organic acid from atmospheric CO_2_, and the organic acid is decarboxylated in mitochondria in the CCC to supply CO_2_ to CCs. In contrast to the NADP-ME type in maize, *Bienertia* operates in NAD-ME type C4 photosynthesis. The CO_2_-capturing PCs have fewer grana stacks than the Rubisco-containing CCs because of CCs’ requirements for reducing power as well as ATP^2,3^.

Biogenesis of SCC4 systems is of interest to plant biologists due to their unique cytoplasmic organization and regulation of photosynthetic genes^6^. Young *Bienertia* chlorenchyma cells have uniform chloroplasts operating in C3 photosynthesis and their cytoplasm is not partitioned^7^. As the chlorenchyma cells mature, vacuoles enlarge to enclose chloroplasts and mitochondria in the cell center and chloroplasts excluded from CCC become PCs^8^. In mature *Bienertia* SCC4 cells, CCs and PCs contain distinct sets of proteins for C4 photosynthesis, and they exchange small molecules through channels composed of cytoplasmic strands. Rubisco large subunit gene in the plastid genome are transcribed specifically in CCs, indicating that transcription in CCs is regulated differently from that in PCs^7^. A mechanism for selective targeting of chloroplast proteins encoded in the nuclear genome to one of the two chloroplast types has been characterized recently. The N-terminal signal peptides of proteins destined for PCs have two components, one that facilitates general entry into the chloroplast and a second one that inhibits import into CCs^9,10^. In agreement with their macromolecular compositions, CCs and PCs exhibit differential ultrastructural characteristics, but there have been no detailed morphometric analyses to show how CCs and PCs diverge from a homogenous pool of chloroplasts in young in *Bienertia* chlorenchyma cells.

Plastids divide via binary fission, and hourglass-shaped dividing proplastids have been observed in electron microscopy (EM) imaging of shoot apical meristem cells^11^. Constriction of the plastid envelope membranes is mediated by two ring complexes, one in the stroma, termed Z-ring, and the other in the cytosol consisting of a dynamin-related protein^12^. The thylakoid membrane is densely populated with massive photosynthetic complexes, and some of them constitute close-packed two-dimensional arrays. It seems a daunting task to bisect piles of thylakoid membranes in grana stacks for chloroplast division but almost none is known about thylakoid fission. However, it has been elusive how thylakoids are partitioned into two daughter chloroplasts during division^13^.

A distinctive feature of the *Bienertia* chloroplast is that large numbers of CCs are packed in the central cytosol; these chloroplasts multiply as chlorenchyma cells develop^7,8^. The *Bienertia* CC cluster provides a unique opportunity to examine thylakoids in dividing chloroplast with transmission electron microscopy (TEM)/electron tomography (ET). Most plastid division in plants happens in meristematic cells in which proplastids have primitive thylakoids. It is challenging to find dividing chloroplasts by TEM because chloroplasts in the leaf divide less frequently than proplastids in the meristematic zone, and leaf cells are large, highly vacuolated^11,14^. It is critical for TEM analysis to acquire unambiguous micrographs of dividing chloroplasts repeatedly in leaf samples of which subcellular structures are preserved close to their native states. The CC division occurs in a restricted volume in the cell center, making it easy to identify many dividing chloroplasts under TEM and capture images of their thylakoids. Furthermore, well-developed thylakoids appear in chlorenchyma cells of young *Bienertia* leaves^15^, facilitating high-pressure freezing fixation of the leaf samples.

ET imaging of cryofixed chloroplast has been an essential approach to determine 3D architectures of the photosynthetic organelle as well as macromolecular structures of its constituents that are close to their native states^16–19^. Taking advantage of these advanced microscopy techniques, we characterized the gradual modifications of thylakoid architectures in CCs and PCs as SCC4 is established in *Bienertia* chlorenchyma cells. We were able to delineate thylakoid growth from inner membrane invaginations that expand over planar thylakoid surfaces to add to a layer in the grana stack. Additionally, the concentrated chloroplasts at the central cytoplasm facilitated EM analyses of chloroplast division. Thylakoid division involved distortion of grana stacks at the constricted site before severing, and displacement of PSII components from the deformed stacks.

## Results

### Morphometric analysis of chloroplasts in maturing *Bienertia* chlorenchyma cells

**Figure 1:**
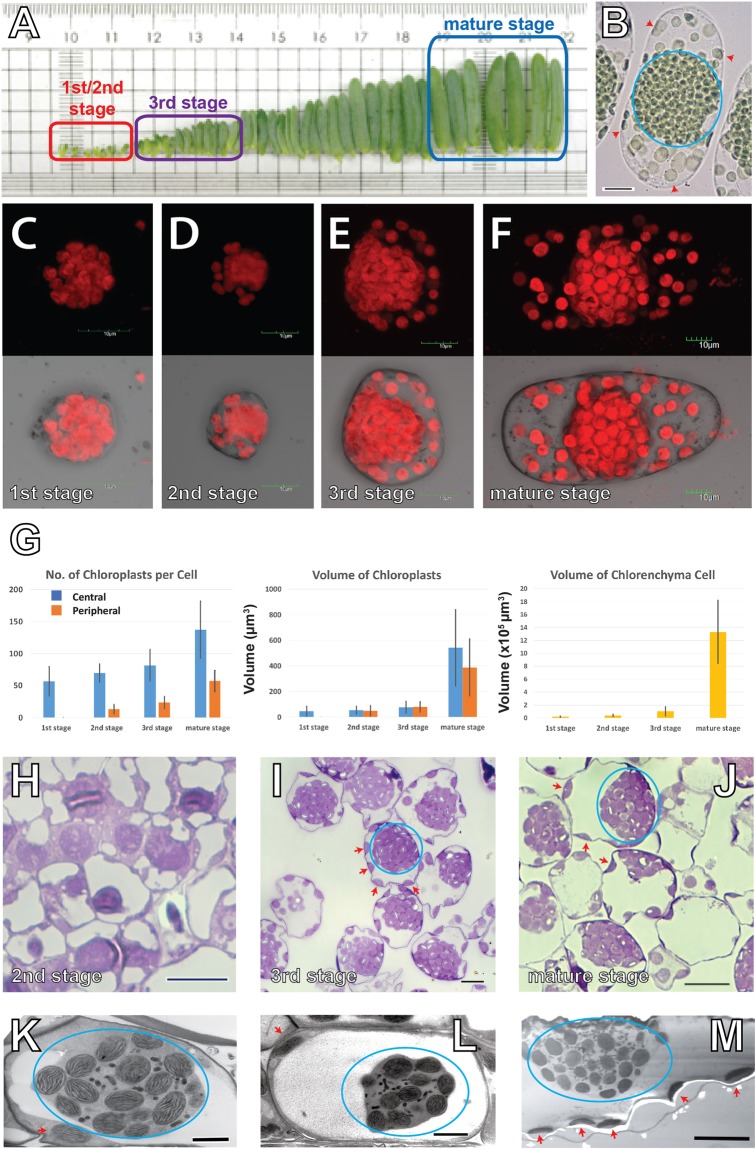
Chloroplast organization in the *Bienertia sinuspersici* chlorenchyma cells. (**A**) Arrangement of *Bienertia* leaves according to their ages from young (left) to old (right). Sources of stages 1, 2, 3, and mature cells are indicated. The 1^st^ stage and the 2^nd^ stage cells are distinguishable only under the microscopes. (**B**) A brightfield light micrograph of a mature chlorenchyma cell. Its CC cluster (blue circle) and PCs (red arrowheads) are marked. Scale bar = 20 µm. (**C–F**) Confocal micrographs of chlorenchyma cells showing chlorophyll autofluorescence at (**C**) 1^st^ stage, (**D**) 2^nd^ stage, (**E**) 3^rd^ stage, and (**F**) mature stage. Scale bars = 10 μm. (**G**) Chloroplast counts (left) and chloroplast sizes (middle) measured from chloroplast autofluorescence. Chlorenchyma cell volumes (right) were estimated from brightfield light micrographs. (**H–J**) Light micrographs of chlorenchyma cells from (**H**) 2^nd^ stage, (**I**) 3^rd^ stage, and (**J**) mature stage cells stained with toluidine blue. Clusters of CCs are denoted with blue circles and PCs are indicated with red arrows in. (**I**,**J)** Scale bars = 10 μm. (**K–M**) Transmission electron micrographs of chlorenchyma cells at (**K**) 2^nd^ stage, (**L**) 3^rd^ stage, and (**M**) mature stage. Small black dots/ovoids in the central cytoplasm inside the blue ovals correspond to mitochondria. Scale bars = 2 µm in panels K and L and 10 µm in panel M.

In order to monitor the maturation process of dimorphic chloroplasts in *Bienertia sinuspersici*, we examined chloroplasts in chlorenchyma cells at 1^st^ stage, 2^nd^ stage, 3^rd^ stage, and the mature stage as defined in Park *et al*.^8^ using confocal laser scanning microscopy and TEM. The 1^st^ stage and 2^nd^ stage cells were sampled from leaves less than 0.3-cm long, 3^rd^ stage cells from leaves 0.4-cm to 0.5-cm long, and mature stage cells from the tip region of leaves 1.5-cm to 2.0-cm long (Fig. 1A). In isolated mature chlorenchyma cells, two groups of chloroplasts were observed**:** densely packed chloroplasts in the central cytosol and chloroplasts scattered in the cell periphery (Fig. 1B).

The 1^st^ stage cells were spherical, and their cytoplasms were occupied mostly by chloroplasts (Fig. 1C). Vacuoles divided the cytosol into central and peripheral regions in older cells, but no large vacuoles were observed in 1^st^ stage cells (Fig. 1D–F). Vacuole development was discerned from 2^nd^ stage, separating some chloroplasts to the cell periphery, and by the 3^rd^ stage, chlorenchyma cells had elongated to take on elliptical shapes and vacuoles segregated PCs from CCs. Cells at the mature stage grew into a pill shape with a corresponding increase in the size and number of chloroplasts (Fig. 1H–M). In TEM micrographs, PCs appear oppressed in the thin cytosol between the vacuole and the plasma membrane (Fig. 1M). Numerous mitochondria were intermixed with chloroplasts in the CCC (Fig. 1K–M).

We were able to estimate cell sizes, cell numbers, and sizes of chloroplasts in the cell at the four stages from confocal laser scanning microscopy data (Fig. 1G). Sizes of mature chlorenchyma cells reached 1,330,971 µm^3^ (s.d. = 487,825 µm^3^, n = 14) with the largest increase between 3^rd^ stage (103,256 µm^3^ (s.d. = 71395 µm^3^, n = 13)) and mature stage (Fig. 1G, left panel). Both CCs and PCs proliferated gradually until the mature stage (Fig. 1G, middle panel). The average chloroplast numbers increased in 1^st^ stage cells to mature cells from 81 (s.d. = 24.64, n = 13) to 137 (s.d. = 44.68, n = 14) for CCs and from 24 (s.d. = 9.82, n = 13) to 56 (s.d. = 16.59, n = 14) for PCs. The volumes of both types of chloroplasts remained similar through stages 1, 2, and 3 (Fig. 1G, right panel). Chloroplasts in mature stage cells were significantly larger than those of 3^rd^ stage cells, however. The volume of CCs increased 7 fold from 76 m^3^ (s.d. = 45.75 m^3^, n = 262) to 541 m^3^ (s.d. = 299.28 µm^3^, n = 303), and the volume of PCs increased 4 fold from 80 m^3^ (s.d. = 41.41 m^3^, n = 218) to 387 m^3^ (s.d. = 222.10 µm^3^, n = 262) during the period.

### Electron tomography analysis of thylakoid assembly

**Figure 2:**
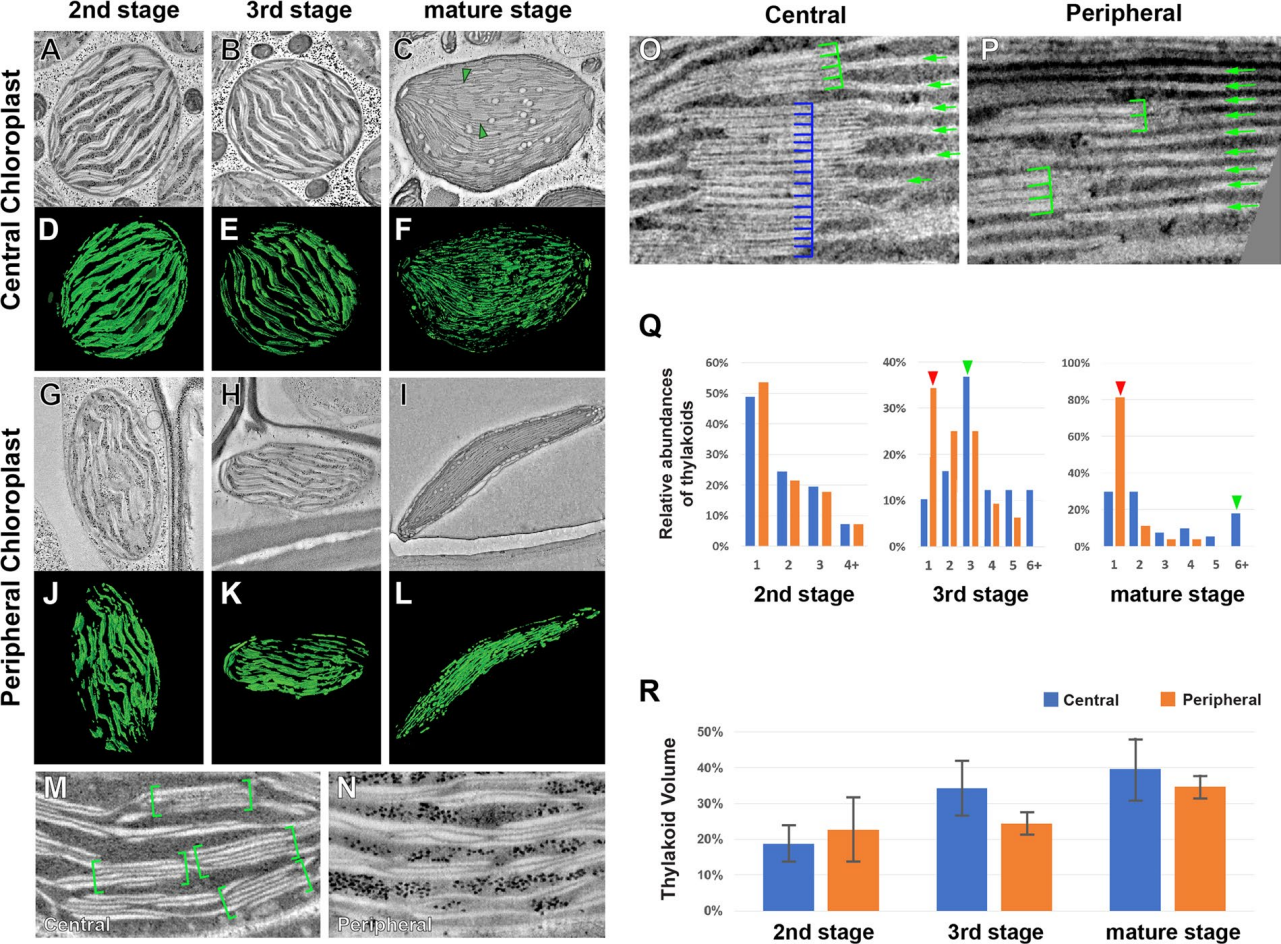
Electron tomography analyses of chloroplast in *Bienertia* chlorenchyma cells. (**A–F**) ET slices of central chloroplasts at (**A**) 2^nd^ stage, (**B**) 3^rd^ stage, (**C**) and mature stage. 3D models of (**D**) 2^nd^ stage, (**E**) 3^rd^ stage, (**F**) and mature stage thylakoids. Grana stacks are marked with green triangles in panel C. (**G–L**) ET slices of peripheral chloroplasts at (**G**) 2^nd^ stage, (**H**) 3^rd^ stage, and (**I**) mature stage. (**J–L**) Thylakoids from (**J**) 2^nd^ stage, (**K**) 3^rd^ stage, (**L**) and mature stage chloroplasts were rendered into 3D models. (**M,N**) ET slice images of a central (**M**) and a peripheral (**N**) chloroplast in 3^rd^ stage cells. (**O**) Image of CC chloroplasts in mature stage cell. The number of layers in a stack varies significantly (4 for the upper stack, green brackets, and 15 for the lower stack, blue brackets). Stacks are interconnected by unstacked stroma thylakoids (green arrows). (**P**) Image of PC chloroplasts. Stacked and unstacked thylakoids are marked with green brackets and arrows, respectively. (**Q**) Abundances of thylakoids determined by number of layers per stack counted in ET slices of *Bienertia* chloroplasts. The difference in grana stack thickness becomes more pronounced in 3^rd^ stage and mature stage chloroplasts. (**R**) Relative volumes occupied by thylakoids in *Bienertia* chloroplasts. The thylakoid volumes were calculated from tomographic models (n = 3) and normalized to stroma volumes of the chloroplasts.

It has been shown by TEM that thylakoid organizations of the CCs and PCs are dissimilar in mature *Bienertia* chlorenchyma cells^7^. We investigated the biogenesis of dimorphic chloroplasts using electron tomography (ET), which allowed three dimensional morphometric analysis of membranous compartments^20^. No ultrastructural differences were apparent in the thylakoid architectures of the two types of chloroplasts in 2^nd^ stage cells, but in 3^rd^ stage chloroplasts, we were able to observe differential architectures in the thylakoids that became even more apparent in mature cells (Fig. 2A–L). The 3^rd^ stage CCs became enriched with grana stacks (2M, green brackets) and short stroma thylakoids connecting the stacks were seen. Grana stacks widened in PCs without obtaining additional layers, making it difficult to identify discrete stacks (Fig. 2N). By the mature stage, the two types of chloroplasts exhibited the structural dimorphism reported previously^15^. Mature CCs were ovoid and grana stacks and unstacked thylakoids were well differentiated in their stroma. Their grana stacks had 2–5 layers and some had as many as 10–11 layers (Fig. 2C,F,O,Q). Mature PCs were flattened into a discus-like shape which appeared as a rod in 2D images (Fig. 2I,L). The thylakoid composition PCs was remarkably skewed toward unstacked stroma thylakoids (Fig. 2P,Q), although there were grana stacks with 2–3 layers (Fig. 2P, green brackets).

We generated 3D models of thylakoid membranes and envelope membranes in the tomograms (Fig. 2D–F,J–L) and calculated volume ratios of thylakoids to their chloroplasts. Relative volumes of thylakoids increased in both types of chloroplasts as they matured (Fig. 2R), indicating that thylakoids proliferate more rapidly than chloroplasts enlarge. Given that chloroplast sizes increased most between the 3^rd^ stage and the mature stage (Fig. 1G), we inferred that thylakoid biogenesis is most active in 3^rd^ stage chloroplasts. From the 3^rd^ stage, volumes of thylakoids in CCs were larger than those in PCs (Fig. 1G).

### Invaginations from the inner envelope membrane and thylakoid assembly

**Figure 3:**
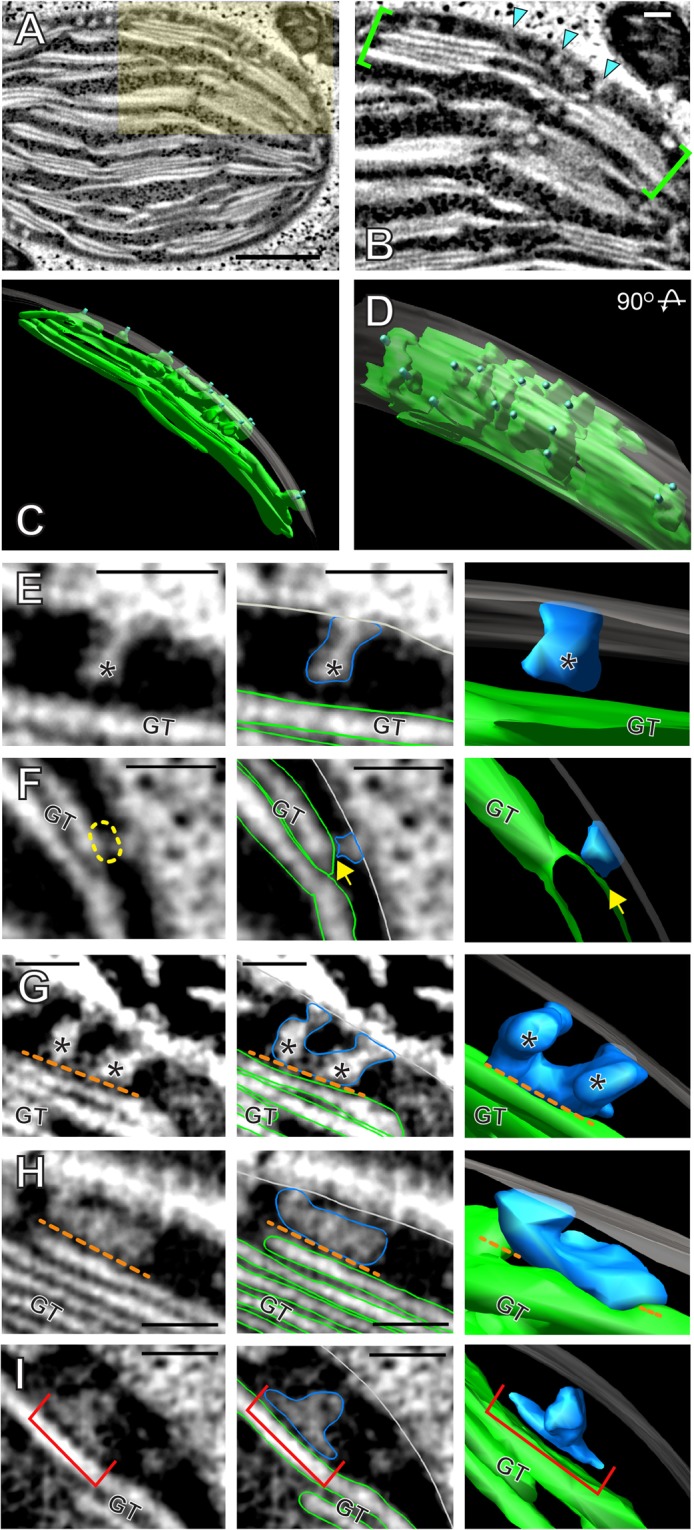
Invaginations from the inner envelope membrane contribute to the growth of thylakoids. (**A**) Electron tomographic slice from a 3^rd^ stage CC. Scale bar = 500 nm. (**B**) The highlighted area in panel A at a higher magnification. Buds and invaginations protrude from the inner envelope membrane (IEM) into the stromal space (cyan arrow heads). Scale bar = 100 nm. (**C,D**) 3D model of the bracketed region in panel B. Panel D shows the model in panel C after 90° rotation. The envelope membrane was rendered semi-transparent. (**E–H**) In these panels, left to right are a tomographic slice; the same slice with IEM outlined in light grey, membrane ingrowths in blue, and existing grana thylakoid (GT) in green; and a 3D model from the tomogram showing (**E**) an ingrowth bud emerging from the IEM. (**F**) An ingrowth appears to be in contact with a GT surface (yellow dashed circle / yellow arrow). (**G**) Two buds of similar sizes that seem to have fused (the orange dotted line marks a gap between the membranes). (**H**) An “L” shaped invagination that lies above an existing grana thylakoid as a separate layer (the orange dotted line marks a gap between the membranes). (**H**) A “T” shaped compartment (red bracket) appears to be an invagination that have separated from IEM. Scale bars = 100 nm.

To investigate how thylakoids assemble in the *Bienertia* chloroplasts, we examined electron tomograms from CCs of 3^rd^ stage cells, the stage of fastest growth. Round membrane outlines were frequently discerned in the stroma close to the envelope and some of them were connected to the envelope membranes (Fig. 3A,B, arrowheads). Similar invaginations were observed in proplastids of cotyledon cells of germinating *Arabidopsis thaliana* seedlings (Liang *et al*.^21^).

We generated 3D models of membrane elements in the vicinity of the ingrowths including the existing thylakoid network and IEM to better understand their relationship (Fig. 3A–D). It is evident that the volume of thylakoid membrane increases in growing chloroplasts. When we arranged IEM invaginations according to their surface areas, we discerned vesicle-shaped ingrowths (Fig. 3E, Movie S1) and more elaborate ingrowths that seemed to have formed by lateral fusion of isolated ingrowths (Fig. 3G, Movie S3). These thylakoid precursors appeared to expand over a grana stack when it encounters its top surface (Fig. 3H, Movie S4). Ingrowths in close proximity with stroma thylakoid (Fig. 3F, arrowhead, Movie S2) were also observed, suggesting that this may constitute a membrane contact site between the IEM and peripheral membranes of the granum. We were able to identify discrete membrane compartments overlaying a thylakoid that seem to have separated from IEM (Fig. 3I, Movie S5), suggesting that the IEM ingrowths can be detached before they merge with the thylakoid system. However, no free-floating vesicles in the stroma were detected.

### Division of chloroplasts within an existing thylakoid network

The number of chloroplasts increased during the *Bienertia* chlorenchyma cell development (Fig. 1G, left panel), and chloroplasts of chlorenchyma cells as early as 2^nd^ stage accumulate grana stacks (Figs. 1 and 2). Grana stacks are packed with large protein complexes of the electron transport chain embedded in the thylakoid membrane, and these stacks could impede separation of daughter chloroplasts. To understand how grana stacks are partitioned in dividing chloroplasts, we examined 2^nd^ stage and 3^rd^ stage *Bienertia* cells with electron microscopy/tomography.

**Figure 4:**
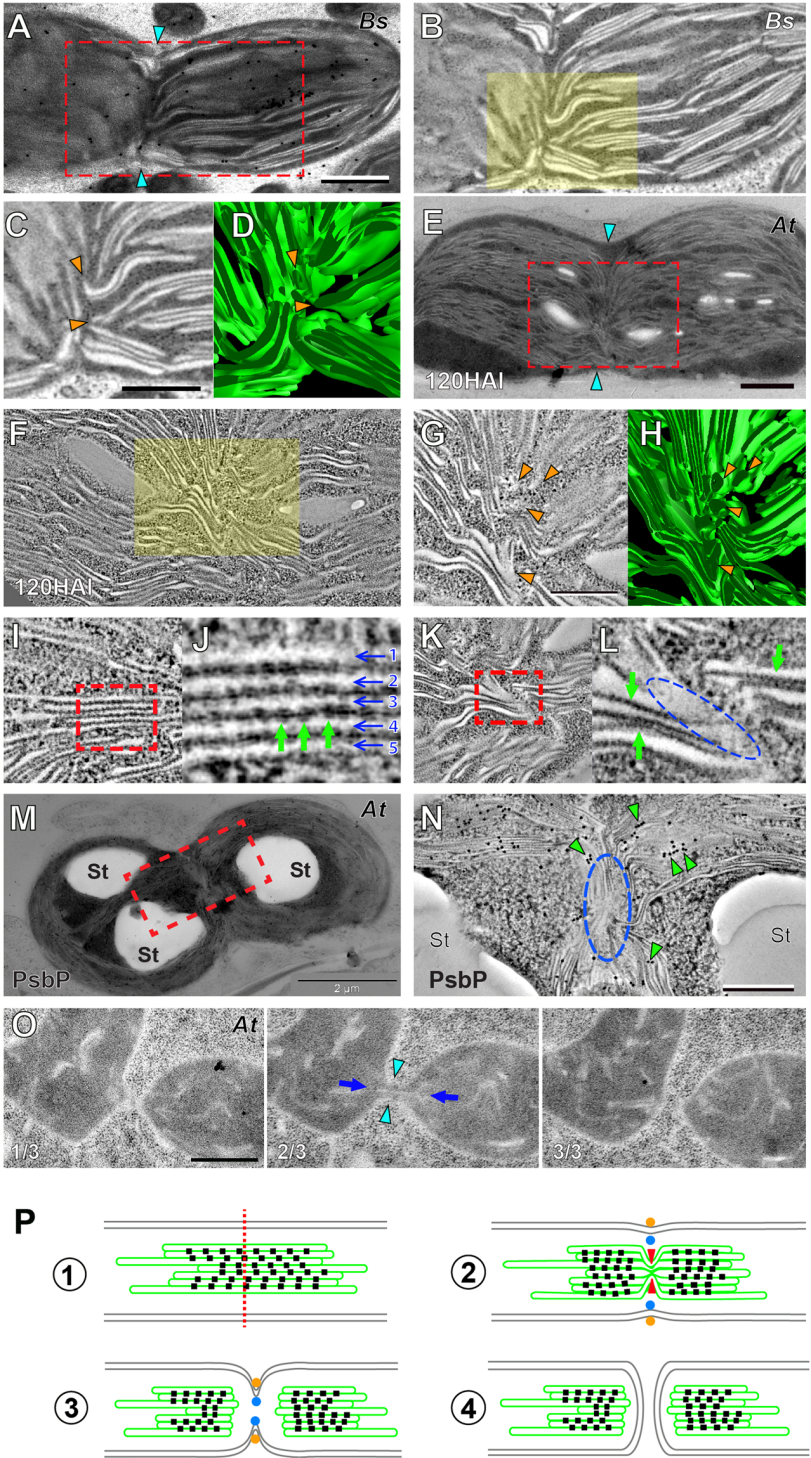
Dividing chloroplasts in *Bienertia* and *Arabidopsis* cells. (**A**) TEM micrograph of a dividing chloroplast in a 3^rd^ stage *Bienertia* cell. The cyan arrows indicate the division furrow. Scale bar = 500 nm. (**B**) ET slice of the boxed area in panel A. (**C**) Magnification of the highlighted area in panel B. The thylakoids have lost the stacked architecture and have been severed (orange arrow heads). (**D**) 3D model of the thylakoids shown in panel C. (**E**) TEM micrograph of a dividing chloroplast in an *Arabidopsis* cotyledon cell at 120 HAI. The cyan arrows indicate the division furrow. Scale bar = 1 µm. (**F**) ET slice of the boxed area in panel E. (**G**) Magnification of the highlighted area in panel F. The thylakoid membranes are sharply kinked (orange arrow heads) in contrast to the planar/stacked thylakoids outside the constricted zone. (**H**) 3D model of the thylakoids in panel G. (**I,J**) ET slice images of thylakoid membranes. Five lamellae of the granum in the boxed area in panel I are indicated by arrows numbered 1–5 in panel J. Pairs of electron dense complexes can be detected on opposite sides of the thylakoid membranes (green arrows). (**K,L**) ET slice images of twisted thylakoids in a dividing chloroplast. Panel L is the high magnification image of the boxed area in panel K. Note the absence of electron-dense complexes in the kinked region (blue dashed oval) as compared to stacked regions around it (green arrows). (**M,N**) Electron micrograph of a dividing *Arabidopsis* chloroplast. PsbP, a subunit of PSII, was localized by immunogold labeling. Panel N is a higher magnification of the boxed area of panel M showing immunogold particles (green arrow heads, 15 nm gold particles). The particles are absent in the squeezed region (blue oval). St: starch particle. Scale bars in **N** = 500 nm (**O**) Three consecutive TEM sections (1/3–3/3) of an *Arabidopsis* meristem cell showing a dividing proplastid. The two daughter plastids are joined via a narrow isthmus (cyan arrowheads in the middle panel). A thin tubule is seen through it (blue arrows). Scale bar = 500 nm. (**P**) Model of the thylakoid fission [**1**] A chloroplast ready to divide; indicated are inner and outer envelope membranes (grey), thylakoid membranes (green), photosystem II (PSII) complexes (black squares), and the plane of division (red dotted line). [**2**] The thylakoid membranes are rearranged as they are ‘pinched’ along the plane of division (red arrowheads) as seen in **E** and **F**. PSII complexes are dislocated from the plane of division. The inner (blue dots) and outer (orange dots) division rings begin squeezing the envelope membranes. [**3**] The thylakoid severing is complete before the division rings separate the chloroplast as seen in (**A**,**B**). [**5**] Two daughter chloroplasts are formed from the division of the mother chloroplast.

Dividing CCs were frequently observed in the central cytoplasmic regions of 2^nd^ stage and 3^rd^ stage cells where dozens of chloroplasts are clustered (Fig. 4A). Very few dividing chloroplasts were seen in the cell periphery under TEM because PCs are rare and scattered over large areas. Dividing chloroplasts were elongated and peanut-shaped due to constriction near the center (arrowheads in Fig. 4A). Interestingly, thylakoids appeared sharply kinked at the division plane at the constricted site (Fig. 4B,C). Electron tomography imaging confirmed that the stack architecture was disrupted. Furthermore, some thylakoids were disjoined as if they had been severed (Fig. 4C,D, arrowheads).

We examined dividing chloroplasts in cryofixed *Arabidopsis* cotyledons to test whether the thylakoid remodeling in dividing *Bienertia* chloroplasts is conserved. Thylakoids began to fold into 2 or 3 layers at 60 hours after imbibition (HAI), and grana stacks constituted the majority of thylakoids in chloroplasts by 120 HAI as previously reported (Liang *et al*.^21^). It was more challenging to detect dividing chloroplasts in 120 HAI *Arabidopsis* cotyledon cells by TEM than it was in *Bienertia* cells.

As in dividing chloroplasts of *Bienertia*, thylakoid membranes in the division zone were highly twisted in 120 HAI *Arabidopsis* chloroplasts (Fig. 4E–H). Electron-dense particles of approximately 10 nm constituting a 2D crystalline array were discerned in appressed membranes of grana stacks (Fig. 4I,J), but these particles were not observed in the kinked thylakoid membranes (Fig. 4K,L). We hypothesize that these particles that have dislocated from the fission sites correspond to PSII. To test this, we localized a subunit of the oxygen-evolving complex of PSII, PsbP, in 120 HAI *Arabidopsis* cotyledon cells using immunogold particles. In dividing chloroplasts, PsbP-specific gold particles were absent from the disrupted thylakoids, indicating that the thylakoid remodeling in dividing chloroplasts involves PSII removal (Fig. 4M,N).

Meristem cells have proplastids, and they do not have extensive grana stacks^22^. We examined dividing proplastids to determine whether their prothylakoids are severed when the ring complexes at the envelope squeeze the envelope membranes. In consecutive sections of an *Arabidopsis* meristem cells where two daughter proplastids are about to separate, we observed a connecting neck and a tubular prothylakoid (Fig. 4O). This observation indicates that prothylakoids are not disconnected in preparation for the shrinking envelope membranes probably because they do not interfere with plastid constriction.

## Discussion

We analyzed development of dimorphic chloroplasts in chlorenchyma cells of *Bienertia sinuspersici* with structural biology approaches. Our results indicate that thylakoids assemble through growth and fusion of inner membrane invaginations and that disjoining of thylakoids precedes constriction of the envelope membranes. The cytoplasmic partitioning through the vacuole development in the early stages separated the chloroplasts into central and peripheral groups. The increases in the cell sizes, numbers of chloroplasts, and chloroplast sizes accelerated in the 3^rd^ stage, culminating in the pill-shape of mature cells with fully diversified chloroplasts. These characteristics of maturation are different from what Koteyeva *et al*. (2016) reported because the stage classification scheme in their research was based on the developmental gradient in a single leaf from the base to the tip^7^.

Our TEM and ET analyses indicated that the 3^rd^ stage is critical in the establishment of the *Bienertia* dimorphic chloroplasts. In 1^st^ stage and 2^nd^ stage cells, no significant ultrastructural variations were detected among chloroplasts. Thylakoids with 2 or 3 layers were abundant in both types of chloroplasts in these early stages. In CCs, beginning in the 3^rd^ stage, the number of layers per thylakoid stack increased and discrete stacks appeared. By contrast, thylakoids spread laterally in PCs, and they lost the layered architecture. That protoplasts from “young cells” are capable of selective protein targeting to the PC was demonstrated by Wimmer *et al*. (2017)^9^; these authors took advantage of this targeting to characterize mechanisms of PC-specific protein accumulation. Considering cell structure and size, 3^rd^ stage cells correspond to “young cells”. It is likely that the morphological variations in the thylakoids in 3^rd^ stage cells result from targeting of proteins involved in thylakoid stacking to CCs and their exclusion from PCs; these proteins include those that stabilize the stack margins such as LHCB subunits and CURT1 family proteins^18,23,24^. Lateral stretching of thylakoids (Fig. 2I–L) and lack of grana-forming proteins in 3^rd^ stage PCs could lead to their distinct thylakoid architecture in the mature stage. Offermann *et al*. (2015) isolated central and peripheral chloroplasts to measure relative abundances of PSII and PSI subunits in the chloroplasts using mass spectrometry analysis^6^. In agreement with the preferential grana stacking in CCs, LHCB proteins mediating membrane stacking are more abundant (up to two-fold) in CCs than PCs.

Thylakoid assembly is the hallmark of chloroplast biogenesis^25,26^. Conversions of proplastids and etioplasts into chloroplasts have been characterized by ET, and these studies led to our current understanding of construction of the photosynthetic apparatus^21,22,27^. We provide evidence for a mechanism of thylakoid assembly involving IEM invaginations that enlarge and become integrated with existing thylakoids. They also formed contact sites with membranes of planar thylakoids (Fig. 3). In *Arabidopsis* proplastids, thylakoid assembly takes place throughout the stroma in a linear order^21,27^. By contrast, the *Bienertia* chloroplast has an elaborate thylakoid network in the stroma and the construction process is confined to the vicinity of the envelope membrane (within 100 nm from IEM). Given that we did not identify vesicle-like carriers in the stroma, roles of vesicle carriers reported earlier from studies in higher plants^28^ seems limited in the *Bienertia* thylakoid assembly.

The intermediate structures derived from IEM resemble what was proposed in Bastien *et al*.^29^. We have detected IEM ingrowth, the formation of flattened invaginations, and the development of contact sites between IEM and planar thylakoids (Fig. 3). These membrane junctions are reminiscent of the endoplasmic reticulum (ER) - chloroplast contact sites. Galactolipids monogalactosyldiacylglycerol (MGDG) and digalactosyldiacylglycerol (DGDG) comprise >75% of the thylakoid membrane and IEM. The rest is composed of two anionic lipids, sulfoquinovosyldiacylglycerol (SQDG) and phosphatidylglycerol (PG)^30^. These four lipids are partly or entirely transferred from ER. The lipid compositions of IEM and thylakoid membrane are broadly similar. Expansion of thylakoid membranes that cannot directly interact with IEM may acquire lipid molecules through the IEM-thylakoid contact sites.

FZL is a dynamin-related protein targeted to the chloroplast. Arabidopsis mutant lines in which *FZL* is inactivated have fragmented thylakoids, suggesting that FZL is involved in thylakoid membrane fusion^21,31^. Recently, Findinier *et al*. demonstrated that FZL in *Chlamydomonas* mediates thylakoid membrane fusion using an ingenious biochemical assay^32^. FZL localizes to thylakoid margins suggesting that grana stacks and stroma lamellae are interlinked through their periphery. FZL orthologs of *Bienertia* could contribute to combining the thylakoid precursors derived from IEM to margins of grana stacks or thylakoid lamellae.

In dividing chloroplasts of *Bienertia* chlorenchyma cells as well as *Arabidopsis* cotyledon cells, kinked thylakoid membranes were seen along the midline connecting the division furrows (Fig. 4). Peanut-shaped chloroplasts that appear to go through fission have been documented before and thylakoids constricted in the neck region have been observed in several land plant species^33,34^. But the unusual thylakoid architecture that we have observed in this study has not been reported previously. We infer that chemical fixation may fail to preserve the intriguing U-shaped thylakoids. It has been demonstrated that high-pressure freezing and subsequent freeze substitution can capture transient and fragile subcellular structures in plant cells that conventional TEM protocols cannot^35,36^. The thylakoid remodeling and splitting visualized in our study benefited from the congregated chloroplast arrangement in growing *Bienertia* chlorenchyma cells and the improved sample preservation methods employed for electron microscopy^37^. It was possible to discern dividing chloroplasts in 120 HAI Arabidopsis cotyledon cell samples, although these were extremely rare, and we examined the displacement of thylakoid membrane proteins in the samples. However, we failed to identify dividing chloroplasts in true leaf cells of *Arabidopsis* seedlings.

Components of the plastid division machinery have been identified and molecular mechanisms of their function have been characterized^38^. Two ring complexes, the DRP5B ring on OEM and the Z-ring inside IEM, assemble at the midzone of a dividing chloroplast to constrict the envelope membrane^39–41^. Linker complexes connecting the two rings on the opposite sides of the envelope regulate formation and contraction of the rings^42^. However, almost no information about thylakoid fission is available^13^. Based on our observations, we speculate that the thylakoid severing machinery bends thylakoid membranes and disrupts the array of PSII-LHCB complexes initiating their relocation, probably by exerting forces (Fig. 4P). Given that thylakoid cutting occurs precisely at the cleavage plane, it is conceivable that assembly and operation of the thylakoid division machinery is coordinated with those of the ring complexes. Because of the distance between the two apparatuses, it is puzzling how the contractile ring complexes at the envelope membranes communicate with the thylakoid division machinery in the stroma. The chloroplast Min system controls position of Z-ring formation and the inner membrane ring seems to trigger subsequent assembly of the linker and outer membrane ring complexes^43^. Establishment of the thylakoid fission machinery could be regulated by the Z-ring. Alternatively, the Min system might control assembly and activation of the thylakoid disjoining directly.

## Conclusions

*Bienertia sinuspersici*, a single-cell C4 plant species, is a useful model organism for studying the development of dimorphic chloroplasts and the selective targeting of nucleus-encoded plastid proteins to central and peripheral chloroplasts. Through light and electron microscopy analyses of *Bienertia* chloroplasts, we delineated the assembly process of thylakoid membranes in a growing chloroplast, observed the gradual gain of grana stacks in the central chloroplasts and their loss in peripheral chloroplasts, and the fission of grana thylakoids in dividing chloroplasts. The observations of thylakoid membranes and the division of chloroplasts presented here diverge from what has been seen in those of proplastids, expanding our understanding of chloroplast biogenesis in higher plants. These observations were made possible by state-of-the-art cryo-preservation and three-dimensional electron microscopy techniques, and investigations of plastids employing these tools in other species are well warranted. Mitochondria are vital in the NAD-ME type C4 photosynthesis occurring in *Bienertia* leaf cells. Further work is needed to characterize the subgroups of mitochondria that associate with the tightly-packed CCs.

## Methods

### Plant growth conditions

*Bienertia sinuspersici* samples were obtained from Dr. Joonho Park (Seoul National University of Science and Technology). The plants were propagated asexually and grown in magenta boxes with full strength Murashige-Skoog (MS) media (1x MS media, 10 mM MES, 10 mM NaCl, 2% sucrose, 0.8% phytoagar, pH 5.8, DW) in a growth chamber (Cat No. MLR-352H-PB, Panasonic, Japan) at a photon flux intensity of 50–100 mol quanta m^−2^ s^−1^ of light under a 16 h/ 8 h light/dark cycle, with temperature of 20–22 °C. Afterwards, Bienertia samples are transferred onto soil and grown in a greenhouse with 100–200 µmol quanta m^−2^ s^−1^ of light where they are watered three times a week with 50 mM NaCl and given a nutritional supplement (1 g/L, NPK 5.1–10–5, BIO-NEX, South Korea) once a week. 2–4 month old plants were used for the experiments. The leaves were then cut and sized to different stages as shown in Fig. 1A. *Arabidopsis* seedlings were grown as described in Lee *et al*.^44^. Briefly, the seeds were germinated and grown on 0.75% phytoagar plates supplemented with ½ MS salt (0.5 g/l, pH 5.7) under continuous light at a photon flux density of 120 mol quanta m^−2^ s^−1^ in a growth chamber at 22 °C before their cotyledons were dissected for high-pressure freezing and electron microscopy experiments.

### Confocal laser scanning microscopy

Isolated chlorenchyma cells were examined using confocal laser scanning microscopes FluoView FV1000 (Olympus, Japan) & TCS SP5 II MP with SMD (Leica Microsystems, Germany) to assess their chlorophyll autofluorescence. Stacked images of the chloroplast in Fig. 1C–F were prepared using FV10-ASW Ver. 3.1 Viewer (Olympus, Japan) and other images were processed using ImageJ (LOCI, University of Wisconsin, USA).

### High-pressure freezing, sample processing, electron microscopy, and electron tomography

High-pressure freezing, freeze substitution, resin embedding, and ultra-microtomy were performed as described^45^. *Bienertia* chlorenchyma tissue and *Arabidopsis* cotyledon tissue were dissected and rapidly frozen with an HPM100 high-pressure freezer (Leica Microsystems, Germany). Two different freeze-substitution protocols were used and processed in an EM AFS2 freeze-substitution machine (Leica Microsystems, Germany). For ultrastructural analysis, the samples were freeze-substituted at −80 °C for 72 h in 2% osmium tetroxide (OsO_4_) dissolved in anhydrous acetone, and excess OsO_4_ was removed by rinsing with precooled acetone. After being slowly warmed up to room temperature over 48 h, tissue samples were separated from planchettes and embedded in Embed-812 resin (Electron Microscopy Sciences, USA). For immunogold labelling samples, anhydrous acetone containing 0.25% glutaraldehyde and 0.1% uranyl acetate was used for freeze-substitution media at −80 °C for 72 h before being slowly warmed up to −45 °C where they were embedded with Lowicryl HM20 resin (Electron Microscopy Sciences, USA) and polymerized under ultraviolet light for 24 h at −45 °C.

The blocks were trimmed and sectioned into 100 nm sections using a diamond knife (Diatome, Switzerland) and an UC7 ultramicrotome (Leica Microsystems, Germany) onto slot grids (Electron Microscopy Sciences, USA) and examined with a Hitachi 7400 TEM (Hitachi-High Technologies, Japan) operated at 80 kV. For ultrastructural analysis, 250 nm thick sections were collected on formvar-coated copper slot grids (Electron Microscopy Sciences, USA) and stained with 2% uranyl acetate in 70% methanol followed by Reynold’s lead citrate (Electron Microscopy Sciences, USA). A 200-kV Tecnai F20 intermediate voltage electron microscopy (Thermo-Fischer, USA) was used to collect tilt series from ±60 at 1.5° intervals in brightfield TEM mode and from ±51° at 1.5° intervals in scanning transmission electron microscopy (STEM) mode. For dual-axis tomography analysis, tilt series around two orthogonal axes were acquired from each section using FEI TEM Tomography and FEI STEM Tomography (Thermo-Fischer, USA)^46^.

The TEM/ET data sets that we produced for this study include 675 electron micrographs (355 *Bienertia*, 320 *Arabidopsis*) of 52 thin sectioned cells, ET reconstruction of 16 chloroplasts (10 *Bienertia*, 6 *Arabidopsis*) with a total of ~5200 slice images, and STEM ET reconstruction of 3 *Bienertia* chloroplasts with a total of 256 slice images. Each micrograph and slice image was analyzed individually. The IMOD (University of Colorado Boulder, USA) software package was used for the reconstruction of tomograms as described in Toyooka and Kang^47^

### Immunogold labelling

The immunogold labelling experiments were performed according to the protocol explained by Kang^48^. Antibodies for PsbP were donated by Kenneth Cline (University of Florida, USA)^49^.

### Automatic segmentation from tomogram

The 3D models of the more complete chloroplast were generated with a total variation-based convex optimization algorithm developed for automatic segmentation of tomograms with incomplete frequency information^50^. The computer codes are written in MATLAB (MathWorks, USA). The data term, a z-axis-dependent threshold, and the regularizing terms were specifically fine-tuned for each tomogram to achieve the most visibly sensible results. The segmented images generated above were then meshed with a MATLAB implementation of the marching cubes algorithm^51^. The meshes were then exported to.stl files with the stlwrite function^52^, which were opened in MeshLab (ISTI-CNR, Italy) for visualization.

### Thylakoid density measurements

Five lines orthogonal to the longest axis of the chloroplast were drawn equidistant from each other. At each line, the thickness of the thylakoid membranes in number of layers was counted using ImageJ (LOCI, University of Wisconsin, USA) along with the thickness of the chloroplast. This data was then quantified using Microsoft Excel (Microsoft, USA).

## Supplementary Information

Supplementary Information 1

Supplementary Information 2

Supplementary Information 3

Supplementary Information 4

Supplementary Information 5
